# Supplementary material for: Pyruvate Dehydrogenase Kinase 1 inhibition mediated oxidative phosphorylation enhancement in cartilage promotes osteoarthritis progression
Source: BMC Musculoskelet Disord. 2023 Jul 20;24:597. doi: 10.1186/s12891-023-06585-6 (PMC10357736; doi:10.1186/s12891-023-06585-6)
Supplement: Supplementary file 1 — Additional file 1. [file 12891_2023_6585_MOESM1_ESM.zip › MF.Gsea.1653627316242/GOMF_TRANSFERASE_ACTIVITY_TRANSFERRING_ALKYL_OR_ARYL_OTHER_THAN_METHYL_GROUPS.html]

Details for gene set GOMF\_TRANSFERASE\_ACTIVITY\_TRANSFERRING\_ALKYL\_OR\_ARYL\_OTHER\_THAN\_METHYL\_GROUPS[GSEA]

|  || Dataset | 55457\_collapsed\_to\_symbols.cls.cls #OA\_versus\_normal.cls.cls #OA\_versus\_normal\_repos |
| Phenotype | cls.cls#OA\_versus\_normal\_repos |
| Upregulated in class | OA |
| GeneSet | GOMF\_TRANSFERASE\_ACTIVITY\_TRANSFERRING\_ALKYL\_OR\_ARYL\_OTHER\_THAN\_METHYL\_GROUPS |
| Enrichment Score (ES) | 0.3402418 |
| Normalized Enrichment Score (NES) | 1.386124 |
| Nominal p-value | 0.03517588 |
| FDR q-value | 0.34188914 |
| FWER p-Value | 1.0 |
Table: GSEA Results Summary

  

Fig 1: Enrichment plot: GOMF\_TRANSFERASE\_ACTIVITY\_TRANSFERRING\_ALKYL\_OR\_ARYL\_OTHER\_THAN\_METHYL\_GROUPS      
 Profile of the Running ES Score & Positions of GeneSet Members on the Rank Ordered List

  

| SYMBOL | TITLE | RANK IN GENE LIST | RANK METRIC SCORE | RUNNING ES | CORE ENRICHMENT || 1 | GSTA4 | glutathione S-transferase alpha 4 [Source:HGNC Symbol;Acc:HGNC:4629] | 216 | 0.808 | 0.0280 | Yes |
| 2 | LTC4S | leukotriene C4 synthase [Source:HGNC Symbol;Acc:HGNC:6719] | 223 | 0.804 | 0.0720 | Yes |
| 3 | GSTK1 | glutathione S-transferase kappa 1 [Source:HGNC Symbol;Acc:HGNC:16906] | 490 | 0.662 | 0.0880 | Yes |
| 4 | GGPS1 | geranylgeranyl diphosphate synthase 1 [Source:HGNC Symbol;Acc:HGNC:4249] | 579 | 0.630 | 0.1161 | Yes |
| 5 | HPGDS | hematopoietic prostaglandin D synthase [Source:HGNC Symbol;Acc:HGNC:17890] | 605 | 0.622 | 0.1486 | Yes |
| 6 | MGST3 | microsomal glutathione S-transferase 3 [Source:HGNC Symbol;Acc:HGNC:7064] | 610 | 0.620 | 0.1826 | Yes |
| 7 | DHPS | deoxyhypusine synthase [Source:HGNC Symbol;Acc:HGNC:2869] | 848 | 0.554 | 0.1949 | Yes |
| 8 | GSTZ1 | glutathione S-transferase zeta 1 [Source:HGNC Symbol;Acc:HGNC:4643] | 974 | 0.523 | 0.2142 | Yes |
| 9 | GSTM3 | glutathione S-transferase mu 3 [Source:HGNC Symbol;Acc:HGNC:4635] | 1025 | 0.512 | 0.2387 | Yes |
| 10 | GSTT1 | glutathione S-transferase theta 1 [Source:HGNC Symbol;Acc:HGNC:4641] | 1129 | 0.491 | 0.2578 | Yes |
| 11 | CLIC2 | chloride intracellular channel 2 [Source:HGNC Symbol;Acc:HGNC:2063] | 1214 | 0.473 | 0.2775 | Yes |
| 12 | GSTP1 | glutathione S-transferase pi 1 [Source:HGNC Symbol;Acc:HGNC:4638] | 1260 | 0.465 | 0.2998 | Yes |
| 13 | LANCL1 | LanC like 1 [Source:HGNC Symbol;Acc:HGNC:6508] | 1507 | 0.428 | 0.3044 | Yes |
| 14 | ALOX5AP | arachidonate 5-lipoxygenase activating protein [Source:HGNC Symbol;Acc:HGNC:436] | 1665 | 0.404 | 0.3145 | Yes |
| 15 | AGPS | alkylglycerone phosphate synthase [Source:HGNC Symbol;Acc:HGNC:327] | 1727 | 0.394 | 0.3316 | Yes |
| 16 | COQ2 | "coenzyme Q2, polyprenyltransferase [Source:HGNC Symbol;Acc:HGNC:25223]" | 1884 | 0.375 | 0.3402 | Yes |
| 17 | FDFT1 | farnesyl-diphosphate farnesyltransferase 1 [Source:HGNC Symbol;Acc:HGNC:3629] | 2538 | 0.299 | 0.3060 | No |
| 18 | MGST2 | microsomal glutathione S-transferase 2 [Source:HGNC Symbol;Acc:HGNC:7063] | 2583 | 0.292 | 0.3188 | No |
| 19 | FDPS | farnesyl diphosphate synthase [Source:HGNC Symbol;Acc:HGNC:3631] | 2681 | 0.282 | 0.3268 | No |
| 20 | FNTA | "farnesyltransferase, CAAX box, alpha [Source:HGNC Symbol;Acc:HGNC:3782]" | 2991 | 0.251 | 0.3167 | No |
| 21 | GSTM5 | glutathione S-transferase mu 5 [Source:HGNC Symbol;Acc:HGNC:4637] | 3123 | 0.238 | 0.3196 | No |
| 22 | GSTM4 | glutathione S-transferase mu 4 [Source:HGNC Symbol;Acc:HGNC:4636] | 3144 | 0.235 | 0.3311 | No |
| 23 | TRMT12 | tRNA methyltransferase 12 homolog [Source:HGNC Symbol;Acc:HGNC:26091] | 3208 | 0.230 | 0.3389 | No |
| 24 | PDSS2 | decaprenyl diphosphate synthase subunit 2 [Source:HGNC Symbol;Acc:HGNC:23041] | 3453 | 0.207 | 0.3314 | No |
| 25 | RABGGTA | Rab geranylgeranyltransferase subunit alpha [Source:HGNC Symbol;Acc:HGNC:9795] | 3491 | 0.203 | 0.3398 | No |
| 26 | GSTA3 | glutathione S-transferase alpha 3 [Source:HGNC Symbol;Acc:HGNC:4628] | 3807 | 0.176 | 0.3250 | No |
| 27 | DHDDS | dehydrodolichyl diphosphate synthase subunit [Source:HGNC Symbol;Acc:HGNC:20603] | 4356 | 0.124 | 0.2892 | No |
| 28 | PTGES | prostaglandin E synthase [Source:HGNC Symbol;Acc:HGNC:9599] | 4399 | 0.121 | 0.2926 | No |
| 29 | DPH1 | diphthamide biosynthesis 1 [Source:HGNC Symbol;Acc:HGNC:3003] | 5835 | 0.006 | 0.1813 | No |
| 30 | GSTM1 | glutathione S-transferase mu 1 [Source:HGNC Symbol;Acc:HGNC:4632] | 5848 | 0.005 | 0.1807 | No |
| 31 | COX10 | cytochrome c oxidase assembly factor heme A:farnesyltransferase COX10 [Source:HGNC Symbol;Acc:HGNC:2260] | 6092 | -0.012 | 0.1624 | No |
| 32 | GSTO1 | glutathione S-transferase omega 1 [Source:HGNC Symbol;Acc:HGNC:13312] | 6275 | -0.025 | 0.1497 | No |
| 33 | NUS1 | NUS1 dehydrodolichyl diphosphate synthase subunit [Source:HGNC Symbol;Acc:HGNC:21042] | 6415 | -0.037 | 0.1409 | No |
| 34 | HMBS | hydroxymethylbilane synthase [Source:HGNC Symbol;Acc:HGNC:4982] | 6582 | -0.048 | 0.1306 | No |
| 35 | NANS | N-acetylneuraminate synthase [Source:HGNC Symbol;Acc:HGNC:19237] | 7530 | -0.121 | 0.0636 | No |
| 36 | GSTT2 | glutathione S-transferase theta 2 (gene/pseudogene) [Source:HGNC Symbol;Acc:HGNC:4642] | 7666 | -0.132 | 0.0604 | No |
| 37 | TSR3 | TSR3 ribosome maturation factor [Source:HGNC Symbol;Acc:HGNC:14175] | 7676 | -0.133 | 0.0671 | No |
| 38 | SRM | spermidine synthase [Source:HGNC Symbol;Acc:HGNC:11296] | 7735 | -0.136 | 0.0701 | No |
| 39 | MAT1A | methionine adenosyltransferase 1A [Source:HGNC Symbol;Acc:HGNC:6903] | 8753 | -0.213 | 0.0028 | No |
| 40 | SMS | spermine synthase [Source:HGNC Symbol;Acc:HGNC:11123] | 8884 | -0.223 | 0.0050 | No |
| 41 | GSTT4 | glutathione S-transferase theta 4 [Source:HGNC Symbol;Acc:HGNC:26930] | 8923 | -0.226 | 0.0146 | No |
| 42 | DPH2 | diphthamide biosynthesis 2 [Source:HGNC Symbol;Acc:HGNC:3004] | 9661 | -0.288 | -0.0268 | No |
| 43 | PDSS1 | decaprenyl diphosphate synthase subunit 1 [Source:HGNC Symbol;Acc:HGNC:17759] | 9750 | -0.296 | -0.0172 | No |
| 44 | RABGGTB | Rab geranylgeranyltransferase subunit beta [Source:HGNC Symbol;Acc:HGNC:9796] | 10431 | -0.364 | -0.0500 | No |
| 45 | PGGT1B | protein geranylgeranyltransferase type I subunit beta [Source:HGNC Symbol;Acc:HGNC:8895] | 10468 | -0.370 | -0.0323 | No |
| 46 | DTWD1 | DTW domain containing 1 [Source:HGNC Symbol;Acc:HGNC:30926] | 10549 | -0.378 | -0.0176 | No |
| 47 | UBIAD1 | UbiA prenyltransferase domain containing 1 [Source:HGNC Symbol;Acc:HGNC:30791] | 11299 | -0.471 | -0.0498 | No |
| 48 | FNTB | "farnesyltransferase, CAAX box, beta [Source:HGNC Symbol;Acc:HGNC:3785]" | 11366 | -0.481 | -0.0282 | No |
| 49 | GSTA1 | glutathione S-transferase alpha 1 [Source:HGNC Symbol;Acc:HGNC:4626] | 11608 | -0.517 | -0.0183 | No |
| 50 | TRIT1 | tRNA isopentenyltransferase 1 [Source:HGNC Symbol;Acc:HGNC:20286] | 11658 | -0.526 | 0.0070 | No |
| 51 | CHM | CHM Rab escort protein [Source:HGNC Symbol;Acc:HGNC:1940] | 11798 | -0.549 | 0.0266 | No |
| 52 | MAT2A | methionine adenosyltransferase 2A [Source:HGNC Symbol;Acc:HGNC:6904] | 12852 | -1.066 | 0.0037 | No |
Table: GSEA details [plain text format]

  

Fig 2: GOMF\_TRANSFERASE\_ACTIVITY\_TRANSFERRING\_ALKYL\_OR\_ARYL\_OTHER\_THAN\_METHYL\_GROUPS      
 Blue-Pink O' Gram in the Space of the Analyzed GeneSet

  

Fig 3: GOMF\_TRANSFERASE\_ACTIVITY\_TRANSFERRING\_ALKYL\_OR\_ARYL\_OTHER\_THAN\_METHYL\_GROUPS: Random ES distribution      
 Gene set null distribution of ES for **GOMF\_TRANSFERASE\_ACTIVITY\_TRANSFERRING\_ALKYL\_OR\_ARYL\_OTHER\_THAN\_METHYL\_GROUPS**
